# Effects of Dietary Guanidinoacetic Acid on the Feed Efficiency, Blood Measures, and Meat Quality of *Jinjiang* Bulls

**DOI:** 10.3389/fvets.2021.684295

**Published:** 2021-07-09

**Authors:** Zengmin Li, Huan Liang, Junping Xin, Lanjiao Xu, Meifa Li, Hanjing Yu, Wenjing Zhang, Yu Ge, Yanjiao Li, Mingren Qu

**Affiliations:** ^1^Key Laboratory of Animal Nutrition in Jiangxi Province, Jiangxi Agricultural University, Nanchang, China; ^2^Laboratory Animal Engineering Research Center of Ganzhou, Gannan Medical University, Ganzhou, China

**Keywords:** Jinjiang bull, growth performance, meat quality, beef growth, feed efficiency, blood measures, antioxidant variables, guanidinoacetic acid

## Abstract

An experiment was conducted to determine the effects of supplementing the diet of *Jinjiang* bulls with guanidinoacetic acid (GAA) on their feed efficiency [feed efficiency were evaluated with feedlot average daily gain (ADG), average daily feed intake (ADFI), and feed-to-gain ratio (F:G)], blood measures, and meat quality. Forty-five *Jinjiang* bulls (24 ± 3 months old and 350.15 ± 30.39 kg by weight) were randomly distributed among five experimental groups (each *n* = 9) and each group was randomly fed with one of five diets (concentrate: roughage ratio of 60:40): (1) control; (2) 0.05% GAA; (3) 0.1% GAA; (4) 0.2% GAA; and (5) 0.4% GAA, respectively. After a 52-days feeding trial, five bulls from the control group and five bulls from the optimal GAA supplementing group were randomly selected and slaughtered for collection of the *longissimus thoracis* (LT) and *semitendinosus* (SM) muscles to determine meat quality. The results showed that dietary GAA improved the ADG, decreased the value of F:G, and affected blood measures and antioxidant variables. Supplementing 0.2% GAA into the diet was optimal for feeding efficiency and most of the measured blood measures. Supplementing 0.2% GAA into the diet increased the a^*^ (redness) values, and b^*^ (yellowness) values, and the amount of creatine kinase (CK), muscle glycogen, creatinine (CRE), and laminin (LN) in LT muscles. However, it decreased the drip loss, L^*^ (lightness) value, and lactate dehydrogenase (LDH) content of LT muscles. Drip loss and shear force decreased in SM muscles, as did the amount of type IV collagen (CV–IV). In conclusion, supplementing 0.2% GAA into the diet could enhance feed efficiency to improve beef growth and meat quality.

## Introduction

Guanidinoacetic acid (GAA) is the only precursor of creatine (Cr) in vertebrates and can be methylated to Cr by guanidinoacetate N-methyltransferase (GAMT) (1–9). Cr acts as a unique energy buffer in the daily life of vertebrates (5–8). As a nutritive feed additive, GAA has been approved by the EFSA (European Food Safety Authority) (10, 11) and the FDA (U. S. Food and Drug Administration) (12) for poultry and swine. It has also been approved by the MOA [Ministry of Agriculture of China (PRC)] (13), in 2014.

GAA is effective at reducing oxidative stress and improving tissue growth and the meat quality of non-ruminant animals (14–16). Spare phosphate radicals from substrate-level phosphorylation and oxidative phosphorylation tend to cause oxidative damage (2–6). Creatine kinases (CK) in the mitochondria and cytoplasm of somatic cells catalyze Cr and spare phosphate radicals to produce phosphocreatine (PCr) (4–8). PCr donates its phosphate to create ATP (adenosine triphosphate), and the resultant energy is used for body growth and various biological activities, acting as an energy buffer. Therefore, supplementing feed with GAA is beneficial for creating Cr to reduce oxidative damage and promote feed efficiency in terms of body growth (2–9, 16). Moreover, Cr, which is converted from GAA, can combine with a hydrate to creatine monohydrate (CMH) or with a phosphate radical to create PCr; this enhances meat quality by binding intracellular hydrates and delaying glycolysis (1–9, 14–16). Finally, GAA could regulate pancreatic secretions, stimulating insulin-like growth factor-1 (IGF-1) release and decreasing glucose concentration in the blood (17–19). Therefore, GAA in the diet is beneficial for animal health, and improves feed efficiency and meat quality. However, there is a limited number of reports on the effect of GAA on feed efficiency, blood and antioxidant measures, and meat quality in beef cattle. Ardalan et al. (20–22), Speer et al. (23), and Li et al. (24) argued that the potential for ruminal degradation would need to be considered if GAA was provided to beef cattle through their diet. Potentially, supplementing a diet with an optimal dosage of GAA could produce effects similar to that of non-ruminant animals (20–24).

Therefore, the objective of our study was to evaluate the effect of GAA to provide new insights into the effects of dietary GAA on feed efficiency, beef growth, blood and antioxidant measures, and meat quality in *Jinjiang* bulls.

## Materials and Methods

### Ethical Statement

These experiments were conducted following the Chinese guidelines for animal welfare. All experimental procedures using laboratory animals were approved by the Animal Care and Use Committee of Jiangxi Agricultural University (Ethics Approval Code: JXAUA01).

### Guanidinoacetic Acid

GAA (99% purity) was acquired from Shandong Yifei Pharmaceutical Co. Ltd. (Shandong, Dezhou, China).

### Animal Treatments and Experimental Diets

This research was undertaken at the beef cattle research and teaching farm, specifically Gaoan Yufeng in Jiangxi province, China. Forty-five *Jinjiang* bulls (24 ± 3 months old, means ± SD) with an initial body weight (IBW) of 350.153 ± 0.39 (means ± SD) kg were randomly distributed among five experimental groups with nine bulls per group. The five experimental groups randomly received GAA supplementation at 0, 0.05, 0.1, 0.2, and 0.4% of DM in the daily basal diet, respectively. The basal diet (Table 1) was designed to support the nutrient needs of protein, energy, vitamins, and minerals, which is consistent with the feeding standards of beef cattle in China, NY/T 815-2004 (Ministry of Agriculture of the People's Republic of China 2004). The feeding trial lasted 45 days with a 7-days adaptation (52 feeding days in total). Diets were offered to the bulls throughout the day. *Jinjiang* bulls were individually housed in single pens (3 ×3.5 m) with feed and water offered *ad libitum* and feed refusals were collected and weighed daily at 07:00.

**Table 1:** Ingredients and nutrient composition of diets for the bulls.

**Items**	**Content (%)**
**Ingredients, %**	
Silage material^a^	40.00
Corn	36.00
Wheat bran	6.00
Soybean meal	15.00
NaHCO_3_	0.60
Premix^b^	2.40
Amount	100.00
**Nutrient composition, %**	
DM	94.02
CP	15.31
NDF	42.14
ADF	21.13
Crude fat	3.52
Ash	9.08
NE_mf_/(MJ/kg)^2^	6.49
P	0.39
Ca	0.49

a *Silage material was rye grass-corn stalks mixed storage (rye grass:corn stalks ratio of 20:80) and its main nutrients were: dry matter 29.38%, crude protein 11.39%, crude fat 0.71%, and crude ash 10.11%*.

b *One kilogram of premix provided the following: Vitamin A 150,000 IU, Vitamin D_3_ 20,000 IU, Vitamin E 3,000 IU, Fe 3 200 mg, Mn 1 500 mg, Zn 2 000 mg, Cu 650 mg, I 35 mg, Se 10 mg, Co 10 mg, Ca 130 g, P 30 g*.

2 *NE_mf_ were calculated values, while others were measured values*.

### Chemical Composition of the Diets

The ratio of concentrate: roughage was 60:40 DM (dry matter). The concentrate consisted of corn, wheat bran, soybean meal, NaHCO_3_, and premix. The roughage was silage material, which was a mix of rye grass and corn stalks (a rye grass: corn stalks ratio of 20: 80).

### Chemical Analyses

Samples were analyzed for DM (method 930.15), ash (method 942.05), ether extract (EE, method 920.39), and crude protein (CP, method 984.13) all according to AOAC (1990) (25). CP was computed as Kjeldahl N^*^6.25. Neutral detergent fiber (NDF), acid detergent fiber (ADF), and acid detergent lignin (ADL) were determined by Van Soest et al.'s method (26). Both ADF and NDF were expressed exclusive of residual ash. Lignin was determined by the solubilization of cellulose with sulfuric acid on the ADF residue (27). NDF was analyzed without the addition of sodium sulfite and heat stable α-amylase. Hemicellulose was calculated as the difference between NDF and ADF. The gross energy content of the diets (MJ/kg DM) was determined in duplicate by bomb calorimetry (7, 25).

### Growth and Feed Efficiency

The IBW and final body weight (FBW) of each bull was measured with an empty stomach at 08:30, at the start and end of the experiment to compute ADG. Daily feed refusals per animal were collected, weighed, thoroughly mixed, and composited before the morning feeding. Daily Feed intakes were determined to calculate ADFI. The value of ADFI: ADG (F:G) was indicated as the feed to gain ratio.

### Blood Sampling

The blood of live *Jinjiang* bulls was collected from the jugular vein before feeding, on the morning of the 50th day of the trial. Three different vacuum blood vessels were used. For each bull, one vacuum blood vessel containing EDTA (ethylene diamine tetra acetic acid) anticoagulant was prepared for the collection of blood for analysis. Another blood sampling was performed using a tube with no anticoagulant to obtain serum. A third blood sampling was collected into the vacuum tube with heparin lithium anticoagulant to prepare plasma. These blood samples were centrifuged at 2000 g and 4°C for 15 min to separate the serum and plasma, then stored at −20°C for the next step of blood biochemical and antioxidant detection.

### Groups in the Slaughter Trial

Considering the best effects of improving feed efficiency, blood measures, and antioxidant variables, the optimal GAA supplementing group was selected from five experimental groups at the end of the feeding trial. The optimal GAA supplementing group and control group (five bulls were randomly selected per group) were prepared for slaughter. Then the selected ten bulls were sacrificed in the slaughterhouse, which is located on the farm.

Slaughter was carried out according to the cattle slaughter operation procedures of China, GB/T19477-2004 (Ministry of Agriculture of the People's Republic of China 2004). Bulls were sacrificed *via* electrical stunning, followed by jugular vein exsanguination. Within 30 min, the LT muscles sampled at the last rib on the left side of the rib cage and the SM muscles sampled on the internal side of the upper tibia of the left hind leg were wrapped with aluminum foil and placed in liquid nitrogen for subsequent tissue chemical analysis.

### Meat Sampling

After slaughter, the sampled muscles [40 g *longissimus thoracis* (LT) and 40 g *semitendinosus* (SM) muscle from each bull] were cryopreserved and taken to a laboratory for homogenizing [referring to Kauffman et al. (28) and Li et al. (29)], after the peripheral muscular membranes had been removed. The other 1500 g of LT and 1500 g of SM in each replicate were vacuum-packed at 4°C for determination of meat quality.

### Blood Cell Analysis

Blood cell analyses were performed with a BC-2800 V et blood analyzer (Mindray Shenzhen, China) at the College of Animal Science and Technology, Jiangxi Agricultural University.

### Blood and Homogenate Biochemical Tests

Blood and homogenate biochemical analyses were conducted with a Cobas 8000 automatic biochemical analyzer (Roche, Basel, Switzerland) at Gannan Medical University. The same measures in blood and tissue biochemical tests were performed with the same reagent kits. Most enzyme activity–aspartate aminotransferase [AST, SFDA Certified No. (2016): 2404192], alanine aminotransferase [ALT, SFDA Certified No. (2016): 2404193], CK [SFDA Certified No. (2015): 2403262], CRE [SFDA Certified No. (2016): 2404188], lactate dehydrogenase [LDH, SFDA Certified No. (2016): 2404206], gamma-glutamyl transferase [GGT, SFDA Certified No. (2016): 2404439], and hydroxybutyrate dehydrogenase [HBDH, SFDA Certified No. (2017): 2400443]–were detected with colorimetric assays (reagent kits: Roche, Mannheim, Germany), while adenosine deaminase [ADA, SFDA Certified No. (2017): 2400845] was determined with a continuous monitoring method (reagent kit: ERKN, Wenzhou, China). Serum urea [BUN, SFDA Certified No. (2017): 2400897] and plasma ammonia [NH_3_, SFDA Certified No. (2017): 2400977], total protein [TP, SFDA Certified No. (2016): 2404452], albumin [ALB, SFDA Certified No. (2016): 2404209], triglycerides [TG, SFDA Certified No. (2017): 2400977], cholesterol [TC, SFDA Certified No. (2016): 2404187], high-density-lipoprotein cholesterol [HDL, SFDA Certified No. (2018): 2400423], and low-density-lipoprotein cholesterol [LDL, SFDA Certified No. (2016): 2403049] were detected with colorimetric assays (reagent kits: Roche, Mannheim, Germany). Since the TP is composed of ALB and globulin (GLB), serum GLB is calculated by subtracting ALB from TP. Plasma homocysteine (HCY) was analyzed with an enzyme cycle assay [reagent kit: MEIKN, Ningbo, China; SFDA Certified No. (2017): 2400332]. Enzymatic assays were applied to the content of CRE [SFDA Certified No. (2013): 2402633] in blood or homogenate, and glucose in blood [GLU, SFDA Certified No. (2017): 2400436] (reagent kits: Roche, Mannheim, Germany). Troponin T (TNT, SFDA Certified No. (2015): 2400203) was examined with elecsys tropnin T kit; free triiodothyronine (FT3) and free thyroxine (FT4) was determined with elecsys FT3 [SFDA Certified No. (2016): 2404449, Roche, Mannheim, Germany] and elecsys FT4 kit (SFDA Certified No. (2016): 2404445; Roche, Mannheim, Germany). These kits are based on electrochemical luminescence detection. Laminin (LN) and type IV collagen (CV-IV) was detected with LN [SFDA Certified No. (2015): 2400484; Autobio, Zhengzhou, China] and a col IV CLIA micro particles kit [SFDA Certified No. (2015): 2400481; Autobio, Zhengzhou, China], using electrochemical luminescence detection. In addition to NH_3_ and HCY being detected using bull blood plasma, other biochemical indicators in the blood were detected using bull serum. Furthermore, muscle glycogen was detected with a muscle glycogen assay kit (CAS No.: A043-1-1, Nanjing Jiancheng Bioengineering Institute, Nanjing, China).

### Antioxidant Index Analyses

The serum content of malondialdehyde (MDA, CAS No.: A003-1), total antioxidant capacity (T-AOC, CAS No.: A015-3-1), reduced glutathione (GSH, CAS No.: A006-2-1), oxidized glutathione (GSSG, CAS No.: A061-1), and the enzyme superoxide dismutase (SOD, CAS No.: A001-3) were determined with commercial kits (Nanjing Jiancheng Bioengineering Institute, Nanjing, China). These antioxidant assays were performed with an Epoch 17121332 enzyme marker (Bio Tek, Vermont, USA) using colorimetric assays at Jiangxi Province Key Laboratory of Animal Nutrition (Nanchang, China).

### Meat Quality

The determination of meat quality followed standard procedures for livestock (NY/T 1333 – 2007; Ministry of Agriculture of the People's Republic of China 2007).

### pH Value

The initial pH (pH _45min_) and final pH (pH _24h_) of LT and SM muscles were measured at 45 min and 24 h postmortem using a pH meter (pHS-3C, Leici, Shanghai, China).

### Meat Color

At 24 h postmortem, a spectrocolorimeter (WSC-S, Shanghai, China) was used to determine meat color, characterized by L^*^ (lightness), a^*^ (redness), and b^*^ (yellowness) values. The freshly cut surface of the meat was exposed in air for 20 min after which the L^*^, a^*^, and b^*^ values from three different locations on each sample of LT and SM were recorded. Each location was measured six times to calculate the means (29, 30).

### Drip Loss

Drip loss was determined with the bagging method. After the peripheral muscular membranes were trimmed off, the LT and SM muscles were cut into meat blocks (2 × 5 × 3 cm). The initial weight of each piece of meat was recorded. One end of each meat piece was hoisted up with iron wire, in the same orientation as the muscle fibers. The meat was then placed in a packaging bag and the bag was filled with air, so that the meat was suspended in the center of the packaging bag, avoiding contact between the meat and the bag. The bag was then sealed and placed in a refrigerator at 4°C, and after 24 h it was reweighed to calculate drip loss (29, 30).

### Cooking Loss

At 24 h post-mortem, the trimmed LT and SM samples (6 × 6 × 4 cm) were sealed within individual vacuum bags after being weighed. While in the bags, the samples were cooked in a water bath at 80°C until the internal temperature of the meat reached 70°C. The meat was then taken out of the bag and cooled to between 0–4°C, and residual moisture was wiped away. The meat was then reweighed to calculate cooking loss.

### Shear Force

The cooked LT and SM samples were then cut into shaped strips (1 × 1 × 3 cm), parallel to the direction of the muscle fibers. The Warner-Bratzler shear force was measured using C-LM3B shear apparatus (Northeast Agricultural University, Harbin, China) with a load cell of 15 kg and a crosshead speed of 200 mm/min. Six replicates of each sample were measured (29, 30).

### Statistical Analyses

Statistical analyses were undertaken using SPSS version 21.0 software (SPSS Inc.). Data are stated as the mean ± SD. One-way ANOVAs and Duncan's multiple range tests were used for the statistical analyses presented in Tables 2–5, to test for differences in average daily weight gain, feed efficiency, blood measures, and antioxidant variables. One-way ANOVAs and Student's *t*-tests were used to compare the control and 0.2% GAA group, presented in Tables 6, 7, after slaughter. Significance was declared at *P* ≤ 0.05 and trend was declared at 0.05 < *P* ≤ 0.10.

**Table 2:** Effects of guanidinoacetic acid on growth and feed efficiency of *Jinjiang* bulls.

**Items**	**Treatment (percentage of dietary DM)**					
	**0% GAA**	**0.05% GAA**	**0.1% GAA**	**0.2% GAA**	**0.4% GAA**	***P*-value**
Initial body weight (kg)	352.13 ± 26.72	343.38 ± 22.27	355.25 ± 40.24	350.38 ± 23.71	349.63 ± 40.83	0.962
Final body weight (kg)	392.50 ± 29.12	392.63 ± 23.74	403.13 ± 43.45	408.63 ± 25.88	397.75 ± 49.33	0.874
ADG (kg/d)	0.780 ± 0.20^b^	0.950 ± 0.15^ab^	0.920 ± 0.16^ab^	1.120 ± 0.11^a^	0.930 ± 0.26^ab^	0.027
ADFI (kg/d)	8.47 ± 0.12^ab^	8.14 ± 0.22^b^	8.10 ± 0.06^b^	8.36 ± 0.72^ab^	8.54 ± 0.08^a^	0.052
F:G	11.69 ± 3.39^a^	8.82 ± 1.58^b^	9.00 ± 1.36^b^	7.60 ± 1.18^b^	10.10 ± 3.77^ab^	0.031

*ADG, average daily gain; ADFI, average daily feed intake; F:G, feed to gain ratio. All traits in this table were analyzed with cattle as the experimental group (n = 9). The results are presented as the mean values ±SD*.

a, b *Significant differences between means within a row are indicated by no common superscript (P < 0.05)*.

**Table 3:** Effects of dietary GAA supplementation on blood analysis of *Jinjiang* bulls.

**Items**	**Treatment (percentage of dietary DM)**					
	**0% GAA**	**0.05% GAA**	**0.1% GAA**	**0.2% GAA**	**0.4% GAA**	***P*-value**
WBC (m/mm^3^)	10.63 ± 0.83	11.46 ± 1.81	10.72 ± 4.46	10.02 ± 1.97	10.00 ± 2.89	0.906
RBC (b/mm^3^)	9.03 ± 1.04	8.89 ± 0.74	8.92 ± 0.75	9.19 ± 1.20	9.31 ± 0.54	0.908
HGB (g/L)	128.83 ± 14.92	131.67 ± 18.34	121.33 ± 12.86	129.00 ± 10.86	126.33 ± 8.02	0.734
HCT (%)	39.85 ± 5.06	41.80 ± 5.68	38.50 ± 4.10	41.73 ± 2.80	40.68 ± 2.62	0.636
MCV (fL)	44.20 ± 1.38	46.88 ± 3.01	43.22 ± 2.48	45.88 ± 4.58	43.73 ± 1.22	0.160
MCH (pg)	14.25 ± 0.50	14.70 ± 0.94	13.58 ± 0.97	14.10 ± 1.19	13.50 ± 0.46	0.127
MCHC (g/L)	323.17 ± 7.08^a^	314.67 ± 7.50^ab^	314.67 ± 7.23^ab^	308.33 ± 8.96^b^	310.00 ± 5.22^b^	0.016
RDW (%)	17.53 ± 1.30	17.08 ± 0.79	17.30 ± 1.02	18.12 ± 1.64	17.75 ± 1.19	0.635
PLT (m/mm^3^)	323.17 ± 150.16	353.83 ± 91.94	354.17 ± 119.30	301.67 ± 154.93	463.83 ± 49.84	0.197
MPV (fL)	5.97 ± 0.33	6.17 ± 0.47	5.92 ± 0.21	6.23 ± 0.32	5.98 ± 0.25	0.396
PDW	15.75 ± 0.14^ab^	15.98 ± 0.38^a^	15.72 ± 0.26^ab^	15.92 ± 0.38^a^	15.45 ± 0.26^b^	0.041
PCT (%)	0.19 ± 0.09	0.22 ± 0.05	0.21 ± 0.07	0.19 ± 0.10	0.28 ± 0.03	0.282

*The results are presented as the mean values ± SD (n = 9)*.

a, b *Significant differences between means within a row are indicated by no common superscript (P < 0.05)*.

*WBC, white blood cell count; RBC, red blood cell count; HGB, hemoglobin concentration; HCT, hematocrit; MCV, mean erythrocyte volume; MCH, mean corpuscular hemoglobin; MCHC, mean corpuscular hemoglobin concentration; RDW, width of erythrocyte volume distribution; PLT, Platelets count; MPV, Mean platelet volume; PDW, Platelet distribution width; PCT, plateletcrit*.

**Table 4:** Effects of dietary GAA supplementation on blood biochemical variables of *Jinjiang* bulls.

**Items**	**Treatment (percentage of dietary DM)**					
	**0% GAA**	**0.05% GAA**	**0.1% GAA**	**0.2% GAA**	**0.4% GAA**	***P*-value**
ALT (U/L)	22.172 ± 0.71^b^	26.205 ± 0.40^ab^	25.003 ± 0.79^ab^	22.671 ± 0.75^b^	27.802 ± 0.68^b^	0.061
AST (U/L)	46.601 ± 2.50^b^	56.607 ± 0.92^ab^	56.203 ± 0.56^ab^	61.808 ± 0.20^b^	64.403 ± 0.36^b^	0.019
GGT (U/L)	16.202 ± 0.39^b^	20.802 ± 0.59^ab^	20.002 ± 0.45^ab^	24.803 ± 0.83^b^	24.207 ± 0.53^b^	0.029
ADA (U/L)	6.330 ± 0.58^b^	6.331 ± 0.15^b^	11.333 ± 0.06^b^	13.331 ± 0.53^b^	9.671 ± 0.53^ab^	0.009
CK (U/L)	95.00 ± 13.91^b^	110.60 ± 36.07^ab^	134.40 ± 25.56^b^	125.60 ± 22.17^ab^	117.80 ± 20.46^ab^	0.160
LDH (U/L)	1174.00 ± 184.51^ab^	1274.17 ± 147.40^b^	1088.67 ± 68.18^b^	1278.17 ± 161.14^b^	1175.83 ± 95.68^ab^	0.121
HBDH (U/L)	1074.80 ± 183.67^ab^	1151.17 ± 139.76^ab^	993.83 ± 62.19^b^	1197.50 ± 157.78^b^	1085.50 ± 99.22^ab^	0.121
TP (g/L)	72.452 ± 0.12^c^	75.934 ± 0.21^bc^	77.302 ± 0.29^b^	79.232 ± 0.10^ab^	81.751 ± 0.67^b^	0.002
ALB (g/L)	39.472 ± 0.48	39.171 ± 0.21	38.122 ± 0.74	38.483 ± 0.66	39.921 ± 0.78	0.733
GLB (g/L)	34.231 ± 0.75^b^	41.082 ± 0.74^b^	40.003 ± 0.87^b^	39.833 ± 0.67^b^	42.081 ± 0.89^b^	0.015
TG (mmol/L)	0.220 ± 0.05	0.210 ± 0.04	0.210 ± 0.05	0.230 ± 0.08	0.220 ± 0.08	0.977
TC (mmol/L)	2.620 ± 0.40	3.000 ± 0.57	2.780 ± 0.54	2.490 ± 0.44	2.970 ± 0.30	0.269
HDL (mmol/L)	2.010 ± 0.10^b^	2.350 ± 0.09^ab^	2.870 ± 0.53^b^	2.530 ± 0.40^ab^	2.770 ± 0.16^b^	0.042
LDL (mmol/L)	1.000 ± 0.22	1.230 ± 0.45	1.120 ± 0.38	0.930 ± 0.25	1.200 ± 0.23	0.434
FT_3_ (pmol/L)	6.690 ± 0.93^b^	6.690 ± 0.11^b^	5.120 ± 0.98^c^	6.380 ± 0.21^ab^	6.550 ± 0.78^ab^	0.031
FT_4_ (pmol/L)	16.430 ± 0.53^b^	15.751 ± 0.68^b^	13.151 ± 0.99^b^	13.530 ± 0.41^b^	14.681 ± 0.08^ab^	0.012
HCY (μmol/L)	7.230 ± 0.60^ab^	6.280 ± 0.59^b^	7.381 ± 0.47^ab^	7.650 ± 0.50^b^	6.300 ± 0.94^b^	0.048
CRE(μmol/L)	144.00 ± 19.41^b^	169.60 ± 13.20^b^	171.80 ± 20.51^b^	148.60 ± 13.72^ab^	156.60 ± 14.52^ab^	0.033
BUN (mmol/L)	5.170 ± 0.79	5.581 ± 0.00	5.171 ± 0.15	5.281 ± 0.01	5.351 ± 0.04	0.949
NH_3_ (μmol/L)	71.752 ± 2.60	67.201 ± 1.37	60.001 ± 4.66	80.606 ± 0.99	66.208 ± 0.38	0.184
GLU (mmol/L)	4.460 ± 0.17^b^	4.440 ± 0.10^b^	4.060 ± 0.15^b^	4.410 ± 0.12^ab^	4.350 ± 0.14^ab^	0.001

*The results are presented as the mean values ± SD (n = 9)*.

a, b *Significant differences between means within a row are indicated by no common superscript (P < 0.05)*.

*ALT, alanine aminotransferase; AST, Aspartate Aminotransferase; GGT, Gamma-Glutamyl Transferase; ADA, adenosine deaminase; CK, creatine kinase; LDH, Lactate Dehydrogenase; HBDH, Hydroxybutyrate Dehydrogenase; TP, total protein; ALB, albumin; GLB=TP-ALB; TG, triglycerides; TC, cholesterol; HDL, high-density-lipoprotein; LDL, low-density-lipoprotein; FT3, free triiodothyronine; FT4, free thyroxine; HCY, serum homocysteine; CRE, serum creatinine; BUN, serum urea; NH_3_, serum ammonia; GLU, blood glucose*.

**Table 5:** Effects of dietary GAA on antioxidant indexes of *Jinjiang* bull serum.

**Items**	**Treatment (percentage of dietary DM)**					
	**0% GAA**	**0.05% GAA**	**0.1% GAA**	**0.2% GAA**	**0.4% GAA**	***P*-value**
MDA (nmol/mL)	3.591 ± 0.83	3.621 ± 0.38	4.772 ± 0.3	4.101 ± 0.89	3.922 ± 0.22	0.837
SOD (U/mL)	25.252 ± 0.23^d^	33.182 ± 0.7^bc^	39.052 ± 0.11^a^	35.414 ± 0.52^ab^	29.376 ± 0.45^cd^	<0.001
T-AOC (U/mL)	4.871 ± 0.09	4.780 ± 0.49	4.700 ± 0.80	4.230 ± 0.78	4.310 ± 0.96	0.603
GSH (μmol/L)	95.733 ± 0.30^c^	94.124 ± 0.57^c^	105.434 ± 0.18^b^	121.329 ± 0.21^a^	90.772 ± 0.28^c^	<0.001
GSSG (μmol/L)	11.120 ± 0.99^a^	10.921 ± 0.35^a^	9.230 ± 0.70^b^	9.720 ± 0.72^b^	10.860 ± 0.43^a^	<0.001
GSH/GSSG	8.670 ± 0.90^c^	8.700 ± 0.78^c^	11.470 ± 0.73^b^	12.490 ± 0.76^a^	8.380 ± 0.55^c^	<0.005

*The results are presented as the mean values ± SD (n = 9)*.

a, b *Significant differences between means within a row are indicated by no common superscript (P < 0.05)*.

*MDA, malondialdehyde; SOD, superoxide dismutase; T-AOC, total antioxidant capacity; GSH, reduced glutathione; GSSG, oxidized glutathione*.

**Table 6:** Effects of dietary GAA on the meat quality of the LT and SM muscles of *Jinjiang* bulls.

**Items**	**Treatment**		***P*-value**
	**0% GAA**	**0.2% GAA**	
***Longissimus thoracis*** **muscle (LT)**			
pH _45min_	6.670 ± 0.35	6.640 ± 0.31	0.890
pH _24h_	5.680 ± 0.20	5.590 ± 0.24	0.550
Lightness, L*	30.811 ± 0.20	28.971 ± 0.09	0.047
Redness, a*	13.380 ± 0.56	15.070 ± 0.79	0.013
Yellowness, b*	2.910 ± 0.24	3.840 ± 0.67	0.036
Drip loss (%)	1.380 ± 0.28	0.860 ± 0.16	0.049
Cooking loss (%)	20.151 ± 0.54	19.821 ± 0.35	0.758
Shear force (N)	29.006 ± 0.01	26.434 ± 0.92	0.480
***Semitendinosus*** **muscle (SM)**			
pH _45min_	6.390 ± 0.41	6.290 ± 0.10	0.642
pH _24h_	5.760 ± 0.33	5.790 ± 0.31	0.900
Lightness, L*	32.452 ± 0.23	32.531 ± 0.47	0.952
Redness, a*	19.881 ± 0.81	19.262 ± 0.85	0.694
Yellowness, b*	5.190 ± 0.69	4.961 ± 0.01	0.685
Drip loss (%)	1.210 ± 0.26	0.770 ± 0.18	0.026
Cooking loss (%)	17.473 ± 0.49	18.535 ± 0.67	0.730
Shear force (N)	47.379 ± 0.65	33.606 ± 0.43	0.109

*The results are presented as the mean values ± SD (n = 5)*.

*P < 0.05 (significant differences between 0.2% GAA vs. 0% GAA)*.

*pH 45 min, initial pH values at 45 min; pH 24 h, final pH values at 24 h after slaughter*.

**Table 7:** Effects of dietary GAA on biochemical metabolites in the meat of *Jinjiang* bulls.

**Items**	**Treatment**		***P-*value**
	**0% GAA**	**0.2% GAA**	
Muscle glycogen (mmol/kg prot)	22.361 ± 0.39	30.270 ± 0.73	<0.001
CRE (μmol/g prot)	3.290 ± 0.11	3.720 ± 0.09	<0.001
CK (U/g prot)	54.291 ± 0.52	59.261 ± 0.12	<0.001
LDH (U/g prot)	5.670 ± 0.24	5.080 ± 0.07	<0.001
ADA (U/mg prot)	7.481 ± 0.49	7.980 ± 0.97	0.547
TNT (ng/mg prot)	39.320 ± 0.62	37.902 ± 0.91	0.317
CV-IV (μg/mg prot)	5.760 ± 0.28	4.490 ± 0.26	<0.001
LN (μg/mg prot)	2.200 ± 0.75	8.212 ± 0.31	0.002

*The results are presented as the mean values ± SD (n = 5)*.

*P < 0.05 (significant differences between 0.2% GAA vs. 0% GAA)*.

*CRE, muscle creatinine; CK, creatine kinase; LDH, lactate dehydrogenase; ADA, adenosine deaminase; TNT, troponin T; CV-IV, type IV collagen; LN, laminin. Only CV-IV was measured in semitendinosus muscles; all the other items were measured in longissimus thoracis muscles*.

## Results

### Growth and Feed Efficiency

As shown in Table 2, there were no significant differences in IBW, FBW, and ADFI among groups (*P* > 0.05). However, the ADG of bulls in the 0.05% GAA group, 0.1% GAA group, 0.2% GAA group, and 0.4% GAA group were higher than that in the control group (*P* < 0.05). Supplementing the diet with 0.2% GAA obtained the maximum ADG of all the groups. The supplementing GAA groups had lower F:G values than did the control group (*P* < 0.05), and supplementing the diet with 0.2% GAA resulted in the lowest F:G value of all the supplementation groups.

### Blood Cell Measures

There were no significant differences in most blood cell measures, such as white blood cell count (WBC), red blood cell count (RBC), hemoglobin concentration (HGB), hematocrit (HCT), mean erythrocyte volume (MCV), mean corpuscular hemoglobin (MCH), width of erythrocyte volume distribution (RDW), platelets count (PLT), mean platelet volume (MPV), and PCT (plateletcrit) among feeding groups (*P* > 0.05) (Table 3). However, mean corpuscular hemoglobin concentration (MCHC) and platelet distribution width (PDW) were affected by GAA supplementation (*P* < 0.05).

### Blood Biochemical Variables

The serum indicators GLU, FT3, and FT4, which could reflect animal energy expenditure, were lower (*P* < 0.05) in the supplemented GAA groups than the control group. The 0.1% GAA group had the largest decline, followed by the 0.2% GAA and 0.4% GAA groups (Table 4). The serum indicators ALT, AST, GGT, and ADA, which are amino acid metabolizing and nucleic acid metabolizing enzymes, were higher (*P* < 0.05) or trending upwards (*P* = 0.06 in ALT) in the supplemented GAA groups compared to the control group. The supplemented 0.2% GAA group was the optimal group for GGT and ADA. Other metabolizing enzymes, such as LDH, HBDH, and CK were not significantly different between groups (*P* > 0.05). Serum TP and GLB were higher in the supplemented GAA groups than the control group (*P* < 0.05) (Table 4), with the 0.4% GAA group being the highest followed by the 0.2% GAA; however ALB did not significantly differ between groups *(P* > 0.05). Among lipid transport and metabolism indicators, HDL was higher in the supplemented GAA groups than the control group (*P* < 0.05), whereas LDL, TG, and TC were not significantly different (*P* > 0.05). In addition, BUN in serum and NH_3_ in plasma were not significantly different between groups (*P* > 0.05). The markers of creatine metabolism, HCY, and CRE were higher (*P* < 0.05) in the supplemented GAA groups than the control group, with the 0.2% GAA group being the highest for HCY (Table 4).

### Antioxidant Variables

Antioxidant indexes including the enzyme activity SOD, the content of GSH, and the ratio of GSH:GSSG were higher in the GAA groups (*P* < 0.05) compared with the control group, while the content of GSSG in the GAA groups was lower (*P* < 0.05) (Table 5). The values of MDA and T-AOC were not significantly different between feeding groups (*P* > 0.05). In terms of regulating antioxidant status, supplemented 0.1% GAA was the optimal group for SOD while the most suboptimal group was 0.2% GAA. Supplemented 0.2% GAA was the optimal group for GSH and the most suboptimal group was supplemented 0.1% GAA. Supplemented 0.2% GAA was the optimal group in terms of the GSH:GSSG ratio while the most suboptimal group was 0.1% GAA. Supplemented 0.1% GAA was the optimal group in terms of GSSG and the most suboptimal group was 0.2% GAA.

### Selected Groups in the Slaughter Trial

The 0.2% GAA and control groups were included in the slaughter trial. Concerning feed efficiency, blood measures, and antioxidant variables, 0.2% GAA was the optimal group (Tables 2–5).

### Meat Quality

Compared to the control group, supplementing 0.2% GAA into the diet increased the a^*^ and b^*^ values and decreased the L^*^ value of LT muscles (Table 6). Meanwhile, supplementing 0.2% GAA into the diet decreased drip loss (*P* < 0.05) and shear force (*P* = 0.109) of SM muscles, and decreased the drip loss (*P* < 0.05) in LT muscles.

### Homogenate Biochemical Variables

Compared to the control group, supplementing 0.2% GAA into the diet clearly promoted muscle glycogen levels along with LN, CK, and CRE (*P* < 0.05) in LT muscles (Table 7). On the contrary, supplementing 0.2% GAA into the diet reduced LDH in the LT muscles (*P* < 0.05). Meanwhile, the TNT content of LT muscles did not differ significantly between groups (*P* > 0.05). However, the CV-IV content of SM muscles was decreased in the 0.2% GAA group compared with the control (*P* < 0.01).

In summary, dietary GAA decreased the ratio of F:G and the serum concentrations of MCHC, GSSG, FT3, FT4, and GLU of the bulls (*P* < 0.05). Dietary GAA increased ADG and PDW values, and serum concentrations of AST, GGT, ADA, TP, GLB, HDL, HCY, CRE, SOD, GSH, and the GSH:GSSG ratio of bulls (*P* < 0.05). Adding 0.2% GAA achieved the best results in the feeding trial. Supplementing feed with 0.2% GAA increased the content of muscle glycogen and CRE, and the a^*^ and b^*^ values in LN muscles (*P* < 0.05). Adding 0.2% GAA into the diet increased the levels of CK and LN in LT muscles (*P* < 0.05). This also decreased LDH and the L^*^ value in LT muscles. Compared to the control, 0.2% GAA decreased the CV-IV content in SM muscles, the drip loss in both LT and SM muscles (*P* < 0.05), and is associated with a decreasing trend in muscle shear force (*P* = 0.109).

## Discussion

### GAA on Growth and Feed Efficiency

Supplementing GAA into the diet has been shown to improve the growth and feed efficiency of beef cattle (24, 31, 32). However, the recommended dose of GAA in beef cattle diets is not uniform. Li et al. (24) and Liu et al. (31) suggested that 0.06–0.09% GAA improved growth and feed efficiency (concentrate: roughage ratio of 50:50) in Angus bulls. Similarly, Qiu et al. (32) reported that 0.05–0.1% GAA (concentrate basis, roughage was ingested under grazing condition) increased beef growth in Hybrid bulls (*Simmental* Cattle × *Xinjiang Brown* Cattle F1 Hybrid). In our study, 0.05–0.1% GAA improved ADG, blood measures and antioxidant capacity in *Jinjiang* bulls, while 0.1–0.4% GAA also enhanced growth and feed efficiency. Supplementing 0.2% GAA into the diet resulted in the highest levels of ADG and minimum F:D ratio, and thus is considered to be the optimal dosage for improving feed efficiency in our study. Combining the results of the aforementioned previous studies (24, 31, 32) with our results for blood measures, ADG and the F:D ratio, it could be inferred that 0.05–0.4% GAA is a suitable supplementation dosage for a bull's diet given subtle differences between different beef breeds, feeding models, and dietary components. This finding is probably related to the potential for ruminal degradation and utilization of GAA in rumen microbiota from different ruminant hosts (20–24).

Bulls have high muscle growth efficiency and are favored by Chinese farmers than cows (18). Mature male animals have a higher metabolic efficiency and daily energy consumption than female animals (2, 4, 24, 28), which is closely related to basal metabolic rate (BMR). High levels of BMR tend to cause oxidative damage and result in “wooden breast” and other myopathies in feeding animals (4, 30, 31). FT3, FT4, and GLU are important variables reflecting BMR. In the current study, the content of serum FT3, FT4, and GLU all decreased with the diet supplemented with GAA, compared with the control group. However, the ADG of the bulls increased while the F:D ratio decreased in the GAA groups. The reason that the beef growth and feed efficiency did not decrease but rather increased could be traced to the regulating effect of GAA. The Cr, which was converted from the dietary GAA, was possibly acting as an energy buffer. In the present study, supplementing 0.2% GAA into the diet promoted the content of muscle glycogen and CK in LT muscles, however it decreased the blood GLU, FT3, and FT4. Perhaps then, GAA decreases a bull's energy expenditure in response to rapid growth and enhancement of the energy storage of its muscles. Meanwhile, the saved energy, which resulted from oxidative stress created during the bull's rapid growth, was possibly used for muscle growth–beef. Therefore, in the current study, AST, GGT (amino acid metabolic enzymes), and ADA (nucleic acid metabolic enzyme), TP and GLB in serum, the LN content (an indicator of fibrosis), and the a^*^ and b^*^ values (redness and yellowness of the beef) all increased in LT muscle while the L^*^ value (brightness of the beef) decreased. It could be inferred that dietary GAA promoted myogenesis while reducing oxidative damage through energy buffering.

### GAA on Energy Buffering and Meat Quality

In our current study, antioxidant variables including SOD, GSH, and GSH:GSSG in the GAA groups were increased over the control group, while the GSSG in the GAA groups was decreased, 0.2% GAA supplementation was the optimal group and 0.1% GAA supplementation was the suboptimal group in terms of enhancing the antioxidant ability of *Jinjiang* bulls. These findings are similar to those of Qiu et al. (29): 0.02–0.1% GAA increased the antioxidant indexes of hybrid bulls (SOD, GSH-px, T-AOC, and CAT) under grazing conditions. The difference between our current study and Qiu et al. (29) may be attributed to the fact that the *Jinjiang* bulls were tethered in cowsheds while the hybrid bulls were kept under grazing conditions. Hybrid bulls probably had higher creatine production and energy utilizing efficiency under grazing conditions with dietary 0.02-0.05% GAA supplementation than did *Jinjiang* bulls with house feeding. In our blood analyses, the optimal group was 0.2% GAA for both MCHC and PDW. GAA may have beneficially affected the water storage properties of blood plasma and the hematopoiesis of the bulls. We found that supplementing 0.2% GAA into the diet improved meat quality (muscle glycogen, CK, LDH, CV-IV, drip loss, shear force, and the L^*^, a^*^ and b^*^ values of beef) through increasing the *Jinjiang* bulls' antioxidant abilities. This result is consistent with the outcomes of supplementing GAA into the feed of other breeds of animals, such as growing-finishing gilts (6), broiler chickens (4, 33), fishes (3), bullfrogs (34–36), and sheep (37–39); nonetheless, the most effective dose differed between species.

In the current study, the drip loss in both LT and SM muscles and the shear force of SM muscles (*P* = 0.109), MCHC in blood, LDH in LT, and CV-IV in SM decreased while the content of muscle glycogen and CK in LT muscle increased. This could be associated with creatine-induced cell super-hydration and delay of glycolysis postmortem, which enhanced meat quality. These outcomes are positive for the consumer. These results are similar to the effects of dietary GAA in Angus bulls (24), sheep (37–39), finishing pigs (1, 2), broiler chickens (33), and bullfrogs (35, 36), demonstrating that supplementing GAA into the diet could produce effects similar for both ruminant and non-ruminant animals.

### The Indicators of GAA on Methylguanidinoacetic and Energy Buffering Reactions

At present, the existing literature (20, 24, 31, 32) and our study revealed that GAA influenced beef growth and feed efficiency in bulls. In addition, GAA influenced body growth and feed efficiency in other ruminants (37–39), and also in poultry and other livestocks (1–9). However, Wang et al. (39) verified that dietary 0.08–0.2% GAA did not influence body growth in gilts; Zhu et al. (6) report the same outcome for 0.045% GAA. The reason for these contrasting results probably lies in the methyl metabolism cycle, because the intake in GAA needed to accept a methyl group to yield Cr through methylation transfer with the help of the enzyme guanidinoacetate N-methyltransferase (GAMT) (24). Gilts need to consume more methyl donors to support high-yields during uterus development and then when giving birth to piglets. Bulls could not only consume plenty of methionine but also deplete a large amount of folic acid as another methyl donor (31). This may be related to the existence of a large number of microorganisms in the digestive tract of herbivores that can synthesize B vitamins. Could there be a GAMT catalytic reaction that occurs in the body after dietary GAA supplementation? This may be evidenced from the serum metabolites HCY and CRE, given that HCY is the end-product of the methylation reaction on GAA (20, 24) and CRE is the end-product of CK catalyzing PCr in an energy releasing reaction (20, 40–44). In our study, we found that dietary GAA increased the HCY content of plasma, and CRE in both serum and LT muscles, indicating a substantial GAMT catalytic reaction and energy buffering effect. However, any kind of feed additive should be added in an appropriate dosage (10–15, 40) to mitigate against adverse effects. Ardalan et al. (20, 23), testified that GAA would result in modest plasma homocysteine (HCY) in cattle assuming that post-ruminal GAA supplementation exceeded 40 g/d. HCY is the cause of atherosclerosis and oxidative damage to blood vessels (20, 23, 41). However, in our feeding trial, the daily pre-ruminal GAA supplementation was 34 g/day in the 0.4% GAA group–the maximum dose group. In addition, in the supplementing GAA feeding trials (with no extra methyl donors such as methionine, folic acid et al.), the value at risk is higher than 18.2 μM in the plasma HCY of cattle (20, 23). In our study, the maximum value of an individual bull's HCY in all GAA supplementing groups was 10.6 μM, and the maximum group average value of HCY was 7.65 μM, which was far lower than the risk range for beef cattle.

## Conclusion

Dietary GAA improved feed efficiency, and the blood and antioxidant measures that enhance beef growth of *Jinjiang* bulls. Supplementing 0.2% GAA into the diet might also improve meat quality. Taking multiple variables into consideration, 0.2% GAA was the optimal dosage for diet supplementation (concentrate: roughage ratio of 60: 40) for *Jinjiang* bulls.

## Data Availability Statement

The original contributions presented in the study are included in the article/supplementary material, further inquiries can be directed to the corresponding author.

## Ethics Statement

The animal study was reviewed and approved by the Committee for the Care and Use of Experimental Animals at Jiangxi Agricultural University (Ethics Approval Code: JXAUA01). Written informed consent was obtained from the owners for the participation of their animals in this study.

## Author Contributions

MQ and HL designed the overall study. ZL, HL, MQ, JX, ML, LX, HY, WZ, YG, and YL performed the experiments. ZL and HL wrote the manuscript. All authors contributed to the article and approved the submitted version.

## Conflict of Interest

The authors declare that the research was conducted in the absence of any commercial or financial relationships that could be construed as a potential conflict of interest.
